# Greater BOLD Variability in Older Compared with Younger Adults during Audiovisual Speech Perception

**DOI:** 10.1371/journal.pone.0111121

**Published:** 2014-10-22

**Authors:** Sarah H. Baum, Michael S. Beauchamp

**Affiliations:** 1 Department of Neurobiology and Anatomy, University of Texas Medical School at Houston, Houston, Texas, United States of America; 2 Vanderbilt Brain Institute, Vanderbilt University, Nashville, Tennessee, United States of America; McGill University, Canada

## Abstract

Older adults exhibit decreased performance and increased trial-to-trial variability on a range of cognitive tasks, including speech perception. We used blood oxygen level dependent functional magnetic resonance imaging (BOLD fMRI) to search for neural correlates of these behavioral phenomena. We compared brain responses to simple speech stimuli (audiovisual syllables) in 24 healthy older adults (53 to 70 years old) and 14 younger adults (23 to 39 years old) using two independent analysis strategies: region-of-interest (ROI) and voxel-wise whole-brain analysis. While mean response amplitudes were moderately greater in younger adults, older adults had much greater within-subject variability. The greatly increased variability in older adults was observed for both individual voxels in the whole-brain analysis and for ROIs in the left superior temporal sulcus, the left auditory cortex, and the left visual cortex. Increased variability in older adults could not be attributed to differences in head movements between the groups. Increased neural variability may be related to the performance declines and increased behavioral variability that occur with aging.

## Introduction

The ability of older adults to understand both auditory-only and audiovisual speech declines with age [1]–[4]. This decline extends to other important cognitive functions, such as memory, visuospatial abilities, and speed of information processing [5]–[8]. Interestingly, performance declines with age are not uniform across multiple trials of the same task. Older adults exhibit much greater variability in performance: on some trials, older adults perform as well as younger adults, but on other trials, older adults perform much worse [9]–[11]. This type of performance decline, referred to as increased intrasubject variability, may be a particularly sensitive measure of age-related changes [12].

We hypothesized that the increased intrasubject variability with age observed in behavioral paradigms should have a neural counterpart. For example, *in vivo* electrophysiology investigations of the visual cortex of experimental animals show increased variability and decreased stimulus specificity with age during viewing of simple visual stimuli [13]–[15]. Increased neural variability in the auditory brain stem response of healthy older adults has been related to deficits in speech in noise perception [16]. Variability on a cognitive motor task has been tied to individual differences in activation and functional connectivity of the dorsal pre-motor cortex [17]. Wide variability has also been observed in the network activation and functional connectivity of posterior default mode and frontal executive networks in a large cohort (1000BRAINS) of healthy older subjects, which may be related to differences in neuropsychological tests of cognitive and motor function [18]. Conversely, other investigations of variability in healthy aging have pointed to the possibility of *decreased* variability with age [19]–[21]. This necessitates continued research to investigate to fully understand the role of response variability in healthy aging.

We used rapid event-related blood-oxygen level dependent magnetic resonance imaging (BOLD fMRI) to measure brain responses to passively-presented audiovisual speech syllables. This allowed us to focus on perceptual processing differences between young and old rather than differences in higher cognitive function and performance. We predicted that repeated presentations of identical speech stimuli would evoke fMRI responses that were more variable in older adults than in younger adults. Neural responses to audiovisual speech were examined using two complementary methods. First, we used a region-of-interest (ROI) analysis focused on the three core areas of the multisensory speech perception network: auditory cortex, visual cortex, and the superior temporal sulcus (STS). Second, we used a voxel-wise analysis to search for differences between older and younger subjects outside of our predefined ROIs.

## Materials and Methods

### 2.1. Ethics Statement

All subjects provided written informed consent and were compensated for their time in accordance with an experimental protocol approved by the Committee for the Protection of Human Subjects of the University of Texas Health Science Center at Houston.

### 2.2. Subjects and exclusion criteria

The young adult cohort consisted of 14 subjects (20–39 years, 6 female, mean age 26.1 years). 24 older subjects were recruited for the study. Five older adult subjects were excluded (two for poor speech identification scores, three for MRI data quality concerns, see details below), leaving 19 total subjects whose data are reported here (53–70 years, 12 female, mean age 63.0 years).

Vision in the older subjects was assessed using a Snellen eye chart. Each eye was tested separately. The range of acuities was 20/20 to 20/70. Hearing in the older subjects were evaluated using a modified Bekesy threshold test at 500 and 2000 Hz [22]. Our subjects had a range of 6.7 dB–30.9 dB for 500 Hz and 7.3–39.4 dB for 2000 Hz, within the normal range (two standard deviations from the mean) for hearing thresholds based on their age [23]. However, speech abilities decline at a different rate than pure audiometric measures later in life [24]. Therefore, we also tested identification of auditory-only and audiovisual syllables. Most subjects scored near ceiling on auditory-only syllable identification (83%−100%, average performance 93%) and audiovisual syllables. Two subjects scored poorly on auditory-only syllable identification (<70%) and were excluded from the analysis. Cognitive function was assessed using the standardized Mini Mental State Examination (MMSE) [25]. All subjects’ scores indicated no decline in cognitive function (scores ranged from 26–30, mean MMSE 28.4, scores 25 out of 30 points or greater indicate normal cognitive function). Two older subjects were excluded for large head movements during fMRI data acquisition (standard deviation of motion regressor >3 mm). One older subject was excluded because an initial analysis of the fMRI data showed response amplitudes more than 3 standard deviations greater than the mean.

### 2.3. Overview of fMRI experiment and analysis

We used two independent methods for fMRI analysis: region-of-interest (ROI) and voxel-wise whole-brain analysis, which give complementary information about brain activity [26], [27]. ROI analysis allows us to examine areas for which we have an *a priori* hypothesis and does not require that data be transformed to a brain template, thus allowing for differences in individual anatomy. Furthermore, it limits Type I errors by limiting the number of statistical tests to a handful of ROIs [28]. However, ROI analyses are blind to effects outside of the predefined ROIs and to functional specialization within ROIs. Therefore, we also performed a voxel-wise analysis to examine activity across the entire brain.

### 2.4. Block-design localizer

A block-design localizer was used to generate the regions-of-interest (ROIs). Each block contained 10 two-second trials, one word per trial, followed by 10 seconds of fixation baseline. Each trial contained a single word from a bank of digital video recordings of 105 single-syllable words (*e.g.* “view”, “door”, “make”) spoken by a female native English speaker. Words were selected from the MRC Psycholinguistic Database [29]. Auditory-only words consisted of the auditory component of each video with a white visual fixation crosshairs and visual-only words consisted of only the visual component of the video recording.

In older adults, the localizer scan series contained six blocks (two auditory-only, two visual-only and two audiovisual blocks in random order). Each block contained a target trial (the word “press”) of the same type (auditory-only, visual-only, or audiovisual) as the other stimuli in the block; subjects were instructed to pay attention to each stimulus and press a response button only during target trials. In younger adults, ten blocks were presented (five auditory-only and five visual-only in random order) with no target trials.

### 2.5. fMRI responses to audiovisual speech syllables

For the main experiment, stimuli were presented in two-second trials in a rapid event-related design. Each trial contained a single audiovisual syllable, consisting of McGurk (auditory “ba”+visual “ga”, auditory “pa”+visual “ka”), non-McGurk incongruent (auditory “ga”+visual “ba”, auditory “ka”+visual “pa”), congruent (“ba”, “ga”, “da”, “pa”, “ka” and “ta”), target (audiovisual “press” in older adults, audiovisual “ma” in younger subjects) and fixation trials (fixation crosshairs only). There was only a single exemplar of each audiovisual syllable, meaning that subjects were exposed to identical stimuli repeatedly. This allowed us to isolate the effects of neural variability. Subjects were instructed to respond with a button press only to target trials and to make no response to all other trials. Behavioral data in the scanner was not collected for two younger subjects. Subjects performed near ceiling on this task (18/19 older adults at 100% accuracy; 10/12 younger adults at 100% accuracy) suggesting a high degree of alertness (no significant difference between groups, t_29_ = 0.6, p = 0.57).

The total length of each video was cropped with digital video editing software (iMovie, Apple Computer) such that each clip started and ended in a neutral, mouth-closed position. Each video stimulus varied in length from 1.7 to 1.8 seconds followed by fixation crosshairs for the remainder of the trial (the crosshairs were always presented in the same screen location as the mouth of the talker visible during other trials in order to minimize eye movements). Prior to the scan, a volume check was conducted for each subject outside the scanner without the presence of scanner noise. Sample videos from the experiment were played and the volume was adjusted so that the volume was “as loud as possible without being uncomfortable or hurting in any way”. After each scan series subjects were asked if they could hear the stimuli presented and if any volume adjustments were necessary.

Subjects viewed audiovisual stimuli presented in a rapid event-related design with slight variations in the number of trials, as follows: older subjects, *n* = 6∶150 audiovisual syllables, 40 target trials; older subjects, *n* = 13∶160 audiovisual syllables, 50 target trials; younger subjects, *n* = 5∶220 audiovisual syllables, 70 target trials; younger subjects, *n* = 9∶200 audiovisual syllables, 80 target trials.

### 2.6. MRI and fMRI analysis

Two T1-weighted MP-RAGE anatomical MRI scans were collected at the beginning of each scanning session with a 3 Tesla whole-body MR scanner (Phillips Medical Systems). The two anatomical scans were aligned to each other and averaged in order to provide maximal gray-white matter contrast. These scans were then used to create a cortical surface model using FreeSurfer [30], [31] for visualization in SUMA [32]. For the fMRI scan series, T2* weighed images were collected using gradient echo-planar imaging (TR = 2000 ms, TE = 30 ms, flip angle = 90°) with in-plane resolution of 2.75×2.75 mm. Auditory stimuli were presented through MRI-compatible in-ear headphones (Sensimetrics, Malden, MA) which were covered with ear muffs to reduce the amount of noise from the scanner. Visual stimuli subtending approximately 20×30 degrees of visual angle were presented on a projection screen with an LCD projector and viewed through a mirror attached to the head coil. Responses to the target trials were collected using a fiber-optic button response pad (Current Designs, Haverford, PA). Analysis of the functional scan series was conducted using Analysis of Functional NeuroImages (AFNI) [33].

#### 2.6.1. fMRI analysis: response amplitude and variability

In order to generate whole brain maps of the amplitude and standard deviation measures at each voxel, we carried out a voxel-wise analysis using the AFNI function *3dDeconvolve,* which uses maximum-likelihood estimation in the context of the generalized linear model (GLM). TENTzero functions were used to estimate the individual hemodynamic response function (using the option *– iresp*) and standard deviation of each response function (using the option *– sresp*) in each voxel for each stimulus type, beginning at stimulus onset and ending 16 seconds later for single syllables and 26 seconds later for blocks of words (the response was constrained to begin and end at zero amplitude). The model functions consisted of independent, piece-wise linear impulse response functions (also known as stick functions) that independently estimated the amplitude of the hemodynamic response at each time point following stimulus presentation. This methodological point is important because it allowed us to estimate the amplitude and standard deviation from the actual response in each individual voxel, not from a fixed hemodynamic response function such as a gamma variate. The use of a fixed function could introduce a confound because of differences in hemodynamic response functions; for instance, if older people had slightly broader hemodynamic response functions, then their deviation from a fixed function would be greater, unrelated to trial-to-trial variability.

For single syllables, we estimated the amplitude of the response as the mean of the response at 4 seconds and 6 seconds after stimulus onset (the peak of the hemodynamic response function). To estimate BOLD variability within each subject for single syllables, the standard deviation at the 4-second and 6-second time points of each impulse response function were averaged to produce a single value per voxel. The brain response to all audiovisual syllables (both response amplitude and variability) was similar, so they were combined for further analysis and only the average across stimulus types (excluding target trials) is reported.

#### 2.6.2. Region-of-interest selection

Data from the whole-brain voxel-wise analysis (*2.6.1*) was first grouped using regions of interest created for each subject individually in native image space. ROIs were selected to target brain areas that are reliably active during multisensory speech perception [34]. A combination of anatomical and functional criteria was used. The anatomic parcellation of the cortical surface was constructed from each individual subject's structural scans with FreeSurfer [35], [36]. Functional criteria were constructed from the independent localizer runs (see section 2.4 for details), eliminating bias [37].

We considered three contrasts when constructing the three regions of interest: auditory words vs. fixation baseline, visual words vs. fixation baseline, and audiovisual words vs. fixation baseline. The STS ROI was defined by finding all voxels in the posterior half of the anatomically parcellated STS that showed a significant response (*t* >2, *p*<0.05) during the localizer (*t* >2 for auditory-only word blocks *vs.* baseline and t >2 for visual-only word blocks *vs.* baseline). For 5 out of 19 older adults, no voxels in the left STS met this criterion, so an alternative criterion was used (*t* >2 for audiovisual word blocks *vs.* baseline). The auditory cortex ROI was defined by finding voxels in the anatomically parcellated transverse temporal gyrus, lateral superior temporal gyrus and planum temporale that were significantly active during the auditory-only blocks (*t* >2 for auditory-only word blocks *vs.* baseline). The extrastriate visual cortex ROI was defined by finding voxels in the anatomically parcellated extrastriate lateral occipitotemporal cortex that were active during the visual-only blocks (*t* >2 for visual-only word blocks *vs.* baseline).

#### 2.6.3. Whole-brain analysis

For the whole-brain voxel-wise analysis, subjects’ individual data were first aligned to the N27 atlas brain [38] using the AFNI function *auto_tlrc*. Blurring kernels of approximately 3–6 mm have been found to be the most sensitive for detecting activation clusters [39]. We chose a 3×3×3 mm FWHM Gaussian kernel to minimize blurring between adjacent ROIs.

To conduct a voxel-wise search for any differences in response amplitude, the average response amplitude (average of the response to all non-target audiovisual speech stimuli relative to fixation baseline at the 4 and 6 second time points) was calculated in each voxel in each subject. *3dttest++* was used to perform an unpaired t-test for every voxel in standard space between the old and young adult groups. The results were mapped from the MRI volume to the cortical surface with *3dSurf2Vol* and masked with the group t-statistic (*t* >2 for the contrast of all audiovisual syllables *vs.* baseline). After the voxel-wise t-test we preformed a clustering technique [40]. This finds only voxels that are significantly active above a particular threshold and spatially contiguous. The probability of finding two voxels above a particular threshold *and* being adjacent is much smaller than the chance of a single voxel above that threshold [41]. Using the AFNI program *slow_surf_clustsim.py*, we estimated that a cluster with a size of 160 mm^2^ would have a corrected p-value of 0.045. A clusterizing filter on the surface (*SurfClust)* was applied and only regions larger than 160 mm^2^ (and *t* >2 for the contrast of all audiovisual syllables *vs.* baseline) are reported.

To conduct a voxel-wise search for differences in intersubject variability, the MATLAB function *vartestn* was used to perform a Bartlett’s multiple sample test for equal variances on the response amplitudes, followed by clusterizing.

To conduct a voxel-wise search for differences in intrasubject variability, an unpaired t-test between groups was performed on the standard deviation of the response at each voxel (*3dttest++*) followed by clusterizing.

#### 2.6.4. Motion correction

Functional data for each subject was first aligned to the averaged anatomical dataset for that subject and then motion-corrected using local Pearson correlation with the AFNI script *align_epi_anat.py* [42]. For each volume, an estimate of the amount of motion in each direction, relative to the reference, was produced. These estimates were used as regressors of no interest in the fMRI analysis. To capture a single value describing the amount of head motion in each subject, the standard deviation of each motion direction across time was averaged across motion directions.

In addition to the standard motion correction steps described above, we also performed the “motion scrubbing” procedure developed by Power and colleagues [43] to further investigate if motion was a potential confound in our main finding of increased intrasubject variability in older adults. First, motion estimates at each time point were calculated in each of the six motion directions (rotational measures: roll, pitch, yaw, and displacement measures: superior, left, and posterior directions). Rotational displacement measures were converted to millimeters using the formula d = R*( ***π*** /180)*r, where R is the rotation in degrees and r is the radius (we used r = 50 mm as prescribed by Power). To express the total amount of motion for each time point in a single value, the absolute value of the displacement in each direction was summed, where the total displacement at the *i*th data point was *D_i_ = |d_α_|+|d_β_|+|d_γ_|+|d_x_|+|d_y_|+|d_z_|*. Then the framewise displacement for the *i*th time point was calculated as *FD_i_ = D_(i-1)_ - D_i_* to express instantaneous head motion. The scrubbing threshold was half of the smallest voxel dimension, as recommended by Power (EPI volumes were collected using a 2.75 mm isotropic voxel, therefore we used a threshold = 1.375 mm). The GLM analysis was then completed a second time, excluding the data points with a framewise displacement exceeding this threshold.

## Results

### 3.1 Responses in the ROIs to audiovisual speech

Our initial analysis focused on three ROIs implicated as critical nodes in the network for multisensory speech perception: the left superior temporal sulcus (STS), the left auditory cortex, and the left extrastriate visual cortex.

#### 3.1.1. Variability of the hemodynamic response in ROIs within subjects

**Figure 1:**
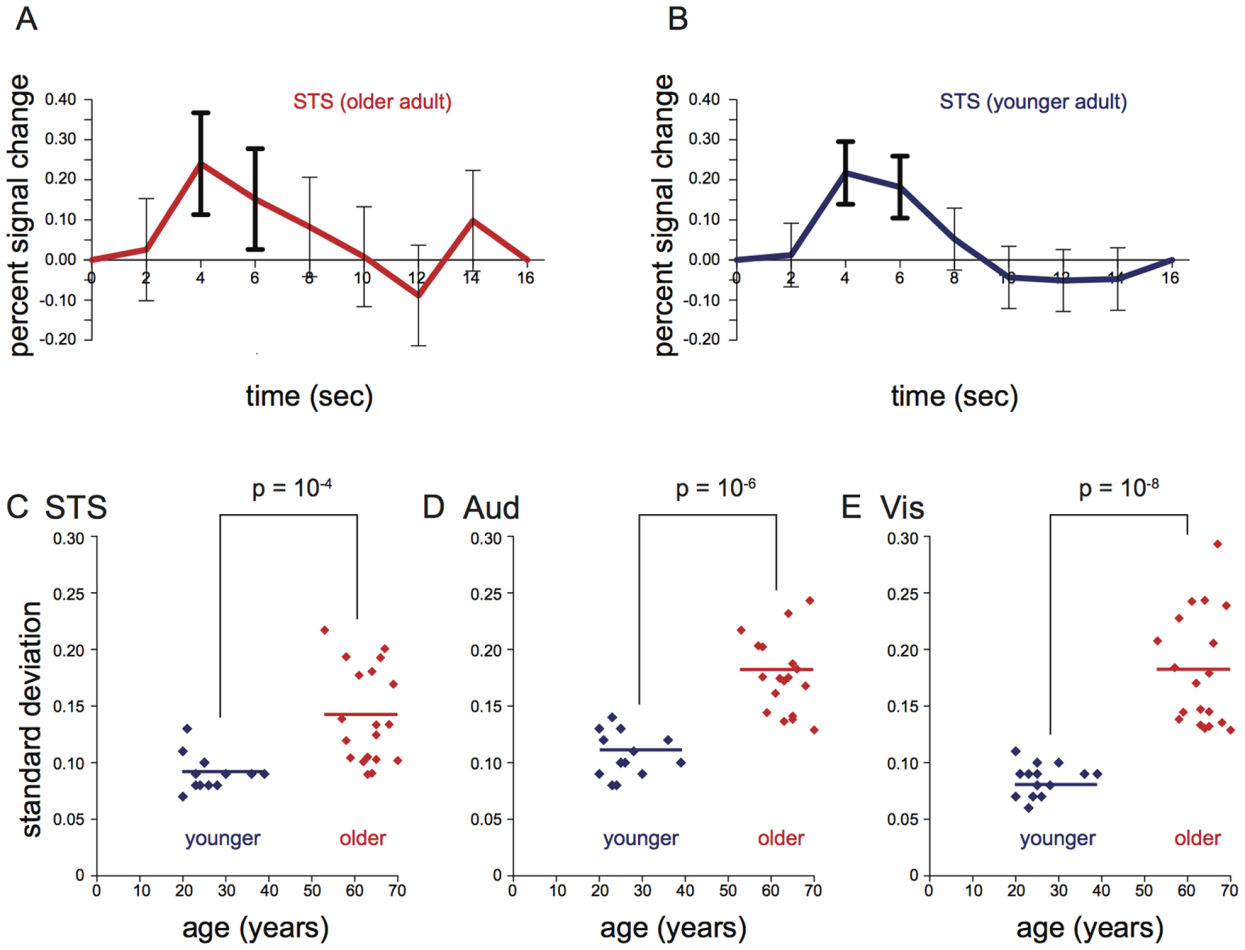
Mean and standard deviation of BOLD responses to audiovisual speech within subjects. A: Hemodynamic response in the left STS of a single older adult (subject JI). Error bars indicate standard deviation of the response within that subject (intrasubject variability) at each time point. The variability at the 4-second and 6-second time points (bold error bars) was used for group analysis. Representative single subject chosen as the subject whose standard deviation was closest to the mean standard deviation for all older subjects. B: Hemodynamic response in the left STS of a single younger adult (subject HU). Representative single subject chosen as the subject whose standard deviation was closest to the mean standard deviation for all younger subjects. C: Scatter plot of age vs. within-subject standard deviation of the STS response. Blue symbols represent younger adults (*n* = 14), red symbols represent older adults (*n* = 19). The lines show the mean of the within-subject standard deviation across each group. The brackets show the results of an unpaired t-test between the within-subject standard deviation in each group. D: Scatter plot of age vs. within-subject standard deviation of the left auditory cortex response. E: Scatter plot of age vs. within-subject standard deviation of the left visual cortex response.

The left STS showed a robust hemodynamic response to audiovisual syllables that was similar in amplitude in individual older and younger subjects. An important behavioral difference in many behavioral paradigms between older and younger subjects is the variability across trials *within* individual subjects (intrasubject variability). To search for a neural correlate of this phenomenon, we calculated the hemodynamic response function in each subject in every voxel and then measured the standard deviation across trials at each time point in the hemodynamic response within each subject (plotting them as error bars around the mean at each time point). As shown in Figure 1A and 1B, while the response amplitudes were similar for individual older and younger subjects, the variability at each time point of the response was much larger in the older subject.

To quantify this difference, we averaged the standard deviation from the peak of the response (4 and 6 seconds after stimulus onset) to produce a single number for intrasubject variability for each ROI for each subject (Figure 1C–E). For each ROI, there was much greater within-subject standard deviation in older subjects (STS: 0.14% in older adults *vs.* 0.09% in younger adults, t_31_ = 4.2, p = 2×10^−4^; auditory cortex: 0.18% *vs.* 0.11%, t_31_ = 5.9, p = 10^−6^; visual cortex: 0.18% *vs.* 0.08%, t_31_ = 7.0, p = 10^−8^).

#### 3.1.2. Mean and standard deviation of the hemodynamic response in ROIs across subjects

An unpaired t-test with percent signal change in the left STS as the dependent measure revealed slightly greater amplitude of mean response in younger adults (0.19% in younger adults *vs.* 0.12% in older adults, t_31_ = 2.1, p = 0.048). There were no significant differences in auditory cortex (0.26% *vs.* 0.24%, t_31_ = 0.5, p = 0.63) or visual cortex (0.16% *vs.* 0.10%, t_31_ = 1.5, p = 0.15).

**Figure 2:**
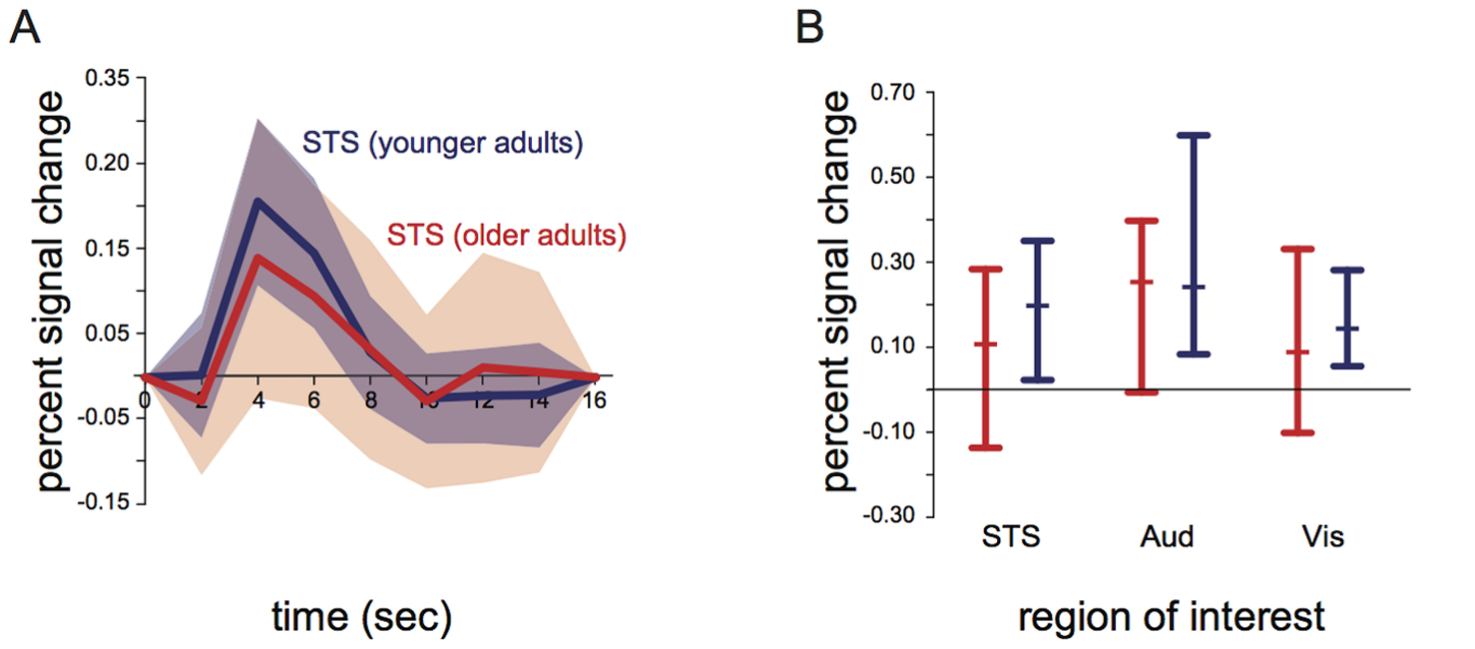
Mean and standard deviation of BOLD responses to audiovisual speech across subjects. A: Average hemodynamic response to audiovisual syllables in the left STS for older adults (red) and younger adults (blue). Shaded region indicates standard deviation of the group response (intersubject variability). B: Response amplitudes in the left STS (STS), left auditory cortex (Aud), and left visual cortex (Vis) across all older adults (red) and younger adults (blue). Error bars show the complete range of data (subjects with maximum and minimum response); middle bar shows median subject.

The standard deviation of the response across subjects was also similar between groups (Figure 2; left STS: SD of 0.08% for younger adults *vs.* 0.12% for older adults, Bartlett’s multiple sample test for equal variances χ^2^_1_ = 2.5, p = 0.12; left auditory cortex: 0.14% *vs.* 0.12%, χ^2^_1_ = 0.7, p = 0.41; left visual cortex: 0.08% *vs.* 0.12%, χ^2^_1_ = 2.3, p = 0.13).

### 3.2. Whole-brain analysis

**Figure 3:**
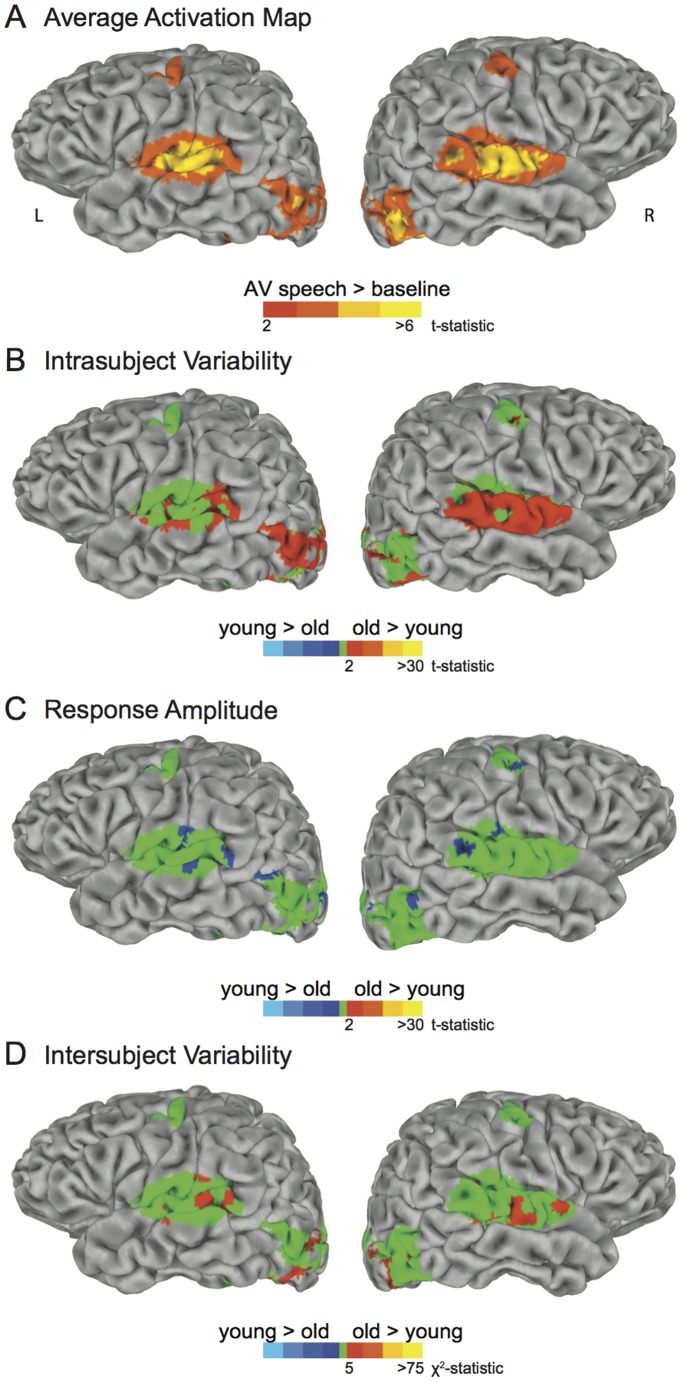
Whole-brain analysis of differences in intrasubject variability, response amplitude, and intersubject variability in older and younger adults. A: Regions that show a significant positive response (*t* >2 for all audiovisual syllables *vs.* baseline) to audiovisual speech in both older and younger adults. L: left hemisphere, R: right hemisphere. B: Differences in intrasubject variability (variability of the amplitude of the BOLD response to audiovisual speech within each subject) between older and younger subjects, masked by active regions in (A). Orange regions indicate areas with greater intrasubject variability in older adults. C: Differences in response amplitude. Green regions indicate no difference in response amplitude; blue regions indicate areas of greater response amplitude in younger adults. D: Differences in intersubject variability (variability of the amplitude of the BOLD response *across* subjects). Orange regions indicate areas with greater response variability in older adults.

Our first set of analyses was limited to our three *a priori* ROIs created using block-design localizers. To overcome this limitation, and to prevent any biases introduced by slight differences in the localizers between old and young subjects, our second set of analyses examined the entire brain. First, we selected all voxels that showed a significant positive response (*t* >2 for all audiovisual syllables *vs.* baseline) to audiovisual syllables across old and young subjects (Table 1 and Figure 3A).

**Table 1:** Regions of activation in response to audiovisual speech in both older and younger adults.

Label	Area (mm^2^)	Peak t-value
R fusiform gyrus, inferior occipital gyrus,middle occipital gyrus *(26,* −*71,* −*6)*	4593	6.2
R fusiform gyrus, inferior occipital gyrus,middle occipital gyrus *(55,* −*21, 2)*	3054	4.3
R superior temporal sulcus and gyrus *(55,* −*21, 2)*	2361	7.4
L superior temporal gyrus *(*−*46,* −*33, 16)*	2315	5.8
L middle occipital gyrus *(*−*23,* −*93, 5)*	560	4.9
R supramarginal gyrus and subcentral gyrus *(49,* −*6, 44)*	219	3.9
Total	13102	

Regions are ranked by area (only clusters greater than 160 mm^2^ are reported) and Talairach coordinates following anatomical label in *(x, y, z)* format are the weighted center of mass of the cluster.

#### 3.2.1. Variability of the whole-brain hemodynamic response within subjects

We calculated the variability of the response *within* each subject at each voxel, and then compared the two groups. Many brain regions showed greater intrasubject variability in older adults (Table 2 and Figure 3B), including left auditory cortex and bilateral extrastriate visual cortex (total area on the cortical surface = 7026 mm^2^; peak t-statistic = 5.8, p = 2×10^−6^). There were no regions where intrasubject variability was greater in younger adults.

**Table 2:** Regions with greater intrasubject variability in older adults compared to younger adults.

Label	Area (mm^2^)	Peak t-value
R superior temporal gyrus *(54,* −*19, 1)*	2341	5.0
L middle and inferior occipital gyri *(*−*38,* −*69,* −*3)*	1943	5.8
L planum temporale *(*−*47,* −*29, 13)*	1697	4.9
R inferior occipital gyrus *(37,* −*61,* −*13)*	882	4.2
R superior temporal sulcus *(45,* −*56, 5)*	163	5.3
Total	7026	

Regions are ranked by area (only clusters greater than 160 mm^2^ are reported) and Talairach coordinates following anatomical label in *(x, y, z)* format are the weighted center of mass of the cluster.

#### 3.2.2. Mean and standard deviation of the whole-brain hemodynamic response across subjects

We compared the amplitude and standard deviation of the response across subjects. Younger adults had greater amplitude of response in right and left visual cortex and right superior temporal sulcus (Table 3 and Figure 3C; total area = 1000 mm^2^; peak t-statistic = 4.3, p = 2×10^−5^; there were no regions where response amplitude was greater in older subjects). Older adults had greater standard deviation of response in bilateral visual cortex and right superior temporal cortex (Table 4 and Figure 3D; total area = 4367 mm^2^; peak Bartlett’s χ^2^_1_ = 29, p = 9×10^−8^; no regions with greater standard deviation in younger adults).

**Table 3:** Regions with greater response amplitude in younger adults compared to older adults.

Label	Area (mm^2^)	Peak t-value
R occipital pole *(13,* −*94, 5)*	302	3.3
R superior temporal sulcus *(47,* −*36, 4)*	273	3.8
L occipital pole *(*−*17,* −*93, 5)*	223	4.3
L subcentral sulcus *(*−*53,* −*21, 13)*	202	3.6
Total	1000	

Regions are ranked by area (only clusters greater than 160 mm^2^ are reported) and Talairach coordinates following anatomical label in *(x, y, z)* format are the weighted center of mass of the cluster.

**Table 4:** Regions with greater intersubject variability in older adults compared to younger adults.

Label	Area (mm^2^)	Peak χ^2^
L inferior occipital gyrus *(*−*30,* −*75,* −*12)*	1819	29
R inferior occipital gyrus *(24,* −*81,* −*8)*	1552	27
R superior temporal gyrus *(62,* −*8, 2)*	438	15
R transverse temporal gyrus *(44,* −*24, 10)*	200	7.8
R fusiform gyrus *(34,* −*49,* −*19)*	194	9.7
L superior frontal gyrus *(*−*1, 0, 56)*	164	27
Total	4367	

Regions are ranked by area (only clusters greater than 160 mm^2^ are reported) and Talairach coordinates following anatomical label in *(x, y, z)* format are the weighted center of mass of the cluster.

### 3.3. Potential confound: differences in head movements between younger and older adults

Our initial analysis used standard techniques for estimation and correction of head motion in the functional data, and our GLM included motion estimates as regressors of no interest. Estimated head motion was small in both groups but greater in older than younger adults (0.48 mm *vs*. 0.32 mm, p = 0.004). The finding of increased BOLD signal variability in older adults remained unchanged after applying a “motion scrubbing” procedure [43]: STS: 0.13% *vs.* 0.09%, t_31_ = 3.4, p = 0.002; auditory cortex: 0.18% *vs.* 0.10%, t_31_ = 5.3, p = 8×10^−6^; visual cortex: 0.17% *vs.* 0.09%, t_31_ = 5.5, p = 1.6×10^−5^. There was no correlation in either group between amount of head motion and intrasubject variability (older adults: r = 0.04, p = 0.88; younger adults: r = 0.16, p = 0.59). An ANCOVA with age group as one factor, head motion as a covariate, and standard deviation of the fMRI response as the dependent variable and revealed no interaction between age group and head motion in any ROI (p>0.68), and the finding of increased intrasubject variability in older adults remained significant (STS: F_1,29_ = 13, p = 0.001; auditory cortex: F_1,29_ = 25, p = 3×10^−5^; visual cortex: F_1,29_ = 34, p = 2×10^−6^). A whole-brain ANCOVA that included the amount of head motion in each subject as a covariate gave results nearly identical to the analysis without the head motion covariate. Discarding the six older adults with the greatest amount of head motion rendered group differences in head movements insignificant (0.39 mm *vs*. 0.32 mm, p = 0.10), but left the main finding of increased intrasubject variability intact (left STS: 0.14% *vs.* 0.09%, t_25_ = 3. 7, p = 0.001; left auditory cortex: 0.18% *vs.* 0.11%, t_25_ = 5.4, p = 10^−4^; visual cortex: 0.17% *vs.* 0.08%, t_25_ = 6.7, p = 5×10^−7^).

## Discussion

We compared brain responses to repeated presentations of identical audiovisual speech in healthy older and younger adults using fMRI. The most important finding was greater intrasubject variability in the older adults: across multiple presentations of identical stimuli, older adults had greater variability in their brain responses than younger adults. This was true across all of the brain areas that responded to audiovisual speech and was confirmed with two independent types of analysis (ROI and whole-brain). We also observed two less robust effects: older adults had smaller mean response amplitudes than younger adults, and the older adult group had a greater standard deviation of the response amplitude (intersubject variability) than younger adults.

### What is the relationship between variability and perception/processing?

Across a variety of behavioral tasks, older adults have worse performance and increased intrasubject variability compared with young adults [11], [44]–[47]. Older adults with mild dementia show more intrasubject variability than healthy age-matched controls [48] and healthy older adults with more trial-to-trial variability showed greater cognitive declines over time [12].

While a link between increased neural (BOLD fMRI) variability and increased behavioral variability is sensible on its face, the precise link between the two is a matter of speculation. With increased neural variability the distribution of responses in a population of neurons to a given stimulus would become wider, which in turn would make it harder for the brain to differentiate between the possible stimuli that evoked the response [49]. Consistent with this idea, decreased stimulus specificity has been observed in single cell recordings of older cats and non-human primates [13]–[15]. Older adults are particularly impaired in perceiving speech if it is embedded in artificially generated noise [2], [4], [50], [51]. The increased noise in the stimulus could exacerbate the effects of increased neural variability, which could be considered “neural noise”. We did not test older subjects using noisy audiovisual speech, the type of speech on which they are most impaired. Therefore, we could not directly compare the increased neural variability we observed in older subjects with the decreased performance of recognizing speech in noise that they are known to have. Future studies using noisy stimuli would be expected to produce poorer performance and reveal differences between subjects correlated with BOLD variability.

Neural variability at early stages of cortical sensory processing might be compounded by neural variability at decision layers higher in the cortical hierarchy. Speech perception involves categorical judgments about the identity of each syllable. Neuronal variability could impair these decisions, an effect that may be even more important than added sensory noise [52]. Neuronal variability may also differentially affect the ability to make both fine and coarse discriminations. Low levels of neuronal variability favor fine discriminations performed at locations in stimulus space in which neuronal selectivity changes rapidly, while high levels of neuronal variability favor coarse discriminations performed at locations in stimulus space where neuronal responses are maximal [53]. Therefore, increased neuronal variability with aging might impair fine discrimination while leaving coarse discriminations relatively intact. Within individual trials, variability in neural responses can allow a population of neurons to encode multiple stimulus attributes. For instance, the mean of the population response may encode the stimulus estimate, while the variability of the population response encodes the uncertainty [54], [55].

### Previous fMRI studies showing greater inter/intrasubject variability in older adults

There have been a number of fMRI studies comparing young and older adults in other tasks, and the preponderance of these studies have reported more variability in older adults, as we observed in our dataset. D’Esposito et al. [56] measured BOLD responses in motor cortex to a bilateral button press cued by the appearance of a briefly presented white circle. Greater intrasubject variability in older adults compared to younger adults was observed; there were no differences in response amplitude and only a small increase in intersubject variability. Huettel et al. [57] measured responses in visual cortex to checkerboard stimuli with no behavioral task. Greater intrasubject and intersubject variability in older adults was observed, with no difference in amplitude of response. Samanez-Larkin et al. [58] found that healthy older adults exhibited suboptimal decision-making on a financial investment task compared to younger adults and also exhibited greater temporal variability in the nucleus accumbens. In contrast to these studies that reported more variability in older adults, Garrett et al. [21] reported *less* neural variability in older adults using a variety of complex cognitive tasks. One possible explanation for this result is that the analysis of Garrett et al. used a measure derived from multivariate voxel pattern analysis (MVPA) to measure variability across all brain voxels. MVPA analyses and traditional univariate analyses such as ours may give conflicting or even contradictory results. The explanation for these discrepancies is a matter of debate [59].

### Potential Confounds

A potential confound in BOLD fMRI studies of older populations is vascular changes with age [60]. However, studies that directly measure neuronal activity also find age-related increases in variability. Anderson et al. [61] presented auditory syllables to healthy older adults and measured the auditory brain stem response, an electrophysiological measure of neuronal activity that is not influenced by the vasculature, and found greater variability in older adults. In a study of non-human primates, single neuron responses in V1 and MT of older monkeys had greater variability than in younger monkeys [62]. These findings suggest that the variability differences in our study have a neuronal component in addition to any possible vascular sources.

Intergroup differences in fMRI studies may also be driven by differences in head movements, especially in studies of resting state functional connectivity [43], [63]. In a task-based study such as ours, averaging the response to multiple stimuli reduces movement effects, since head movements and stimulus presentation are independent. We used five different methods to account for differences in head motion, and found no effect on our main results. Consistent with these analyses, two previous studies (Huettel et al. [57] and D’Esposito et al. [56]) did not find a correlation between head motion and BOLD signal variability.

## Conclusion

Our most robust finding was of greater intrasubject variability in older adults. This finding was true in both the ROI and whole-brain analysis. Among the physical changes to the aging brain, a decrease in myelination has been observed [64], [65]. These decreases in white matter integrity could lead to increases in neuronal variability by preventing neurons from firing consistently even with the same sensory input. Better understanding the neural sources of this variability and its behavioral consequences may help in designing strategies to ameliorate declines in speech perception, one of our most important cognitive functions.
